# Effects of Sanda Sports Training on Cognitive–Motor Control Based on EEG and Heart Rate Sensors: A Coupled ERP and HRV Analysis

**DOI:** 10.3390/s25216558

**Published:** 2025-10-24

**Authors:** Ziwen Ning, Jiayi Zhao, Chuanyin Jiang, Haojie Li, Haidong Jiang, Tianfen Zhou

**Affiliations:** 1 School of Wushu, Shanghai University of Sport, Shanghai 200438, China; ningziwen534@163.com (Z.N.); jcy@sus.edu.cn (C.J.); jianghaidong@sus.edu.cn (H.J.); 2 School of Psychology, Shanghai University of Sport, Shanghai 200438, China; zhaojiayi94@163.com; 3 School of Exercise and Health, Shanghai University of Sport, Shanghai 200438, China; 202121070037@mail.bnu.edu.cn

**Keywords:** sanda athletes, event-related potentials, heart-rate variability, conflict adaptation, neuro-cardiac coupling

## Abstract

**Objective**: To investigate whether prolonged Sanda combat experience improves cognitive–motor control via neuro-cardiac coupling. **Methods**: Nineteen national-level Sanda athletes and nineteen matched controls completed a color-word Stroop task while concurrent EEG and ECG were recorded. The conflict adaptation effect (CAE), which refers to the ability to adjust cognitive control in response to conflicting stimuli, was compared between groups, along with P600 and LSP amplitudes and heart rate variability (RMSSD, HF); mediation analysis examined vagal recovery as a pathway. **Results**: Athletes responded faster and showed a larger CAE than controls (*p* < 0.001). ERP analyses revealed larger CAE-related P600 and LSP amplitudes in athletes (*p* < 0.05), with LSP amplitude inversely correlating with behavioral CAE (*p* < 0.05). Post-task vagal rebound (ΔRMSSD and ΔHF) was significantly greater in athletes (*p* < 0.05), and ΔRMSSD positively correlated with CAE (*p* < 0.05). Mediation analysis confirmed that vagal recovery partially mediated the association between Sanda experience and improved cognitive–motor control (*p* < 0.05). **Conclusions**: Sanda training enhances cognitive–motor control by accelerating parasympathetic recovery and optimizing neural conflict processing, providing evidence for an integrated exercise–cognition–autonomic nervous system coupling model.

## 1. Introduction

WuShu Sanda is a high-intensity combat sport that combines kicking, striking, and throwing techniques, requiring athletes to respond quickly and execute precise movements in rapidly changing combat environments [1]. Previous studies have shown that combat sports (such as boxing and taekwondo) can significantly improve an individual’s neural regulation abilities, including enhancing motor cortex excitability, optimizing sensory–motor integration, and improving reaction inhibition functions [2,3]. For example, athletes who have engaged in long-term combat training exhibit superior performance in limb coordination, dynamic balance, and decision-making speed compared to the general population, suggesting that such sports may have a unique shaping effect on the brain’s cognitive–motor control system [4]. However, despite WuShu Sanda being a representative of traditional Chinese combat sports, quantitative research on its training effects in the field of neuroscience remains limited. Existing research has primarily focused on other martial arts disciplines, with limited empirical evidence on the neural mechanisms and cognitive–motor control effects of Sanda. Moreover, research on how the absence of Sanda combat experience influences the conflict adaptation effect is lacking, and the integration of ERP and HRV studies remains underexplored. Systematic exploration using objective physiological indicators (such as electroencephalography and heart rate variability) is urgently needed [5,6].

Cognitive–motor control refers to the brain’s higher-order regulatory capacity in integrating sensory information and motor execution processes, directly influencing an individual’s reaction speed, movement accuracy, and environmental adaptability [7,8]. Previous studies have found that this ability is not only a core element of competitive sports performance but is also closely related to motor learning, injury prevention, and rehabilitation in daily life [9,10]. For example, high-level athletes demonstrate superior cognitive flexibility and motor inhibition in conflict tasks, and motor interventions (such as aerobic training and coordination training) have been shown to enhance functional connectivity between the prefrontal cortex and motor cortex [11,12]. As a sport that combines high-intensity physical exertion with complex tactical decision-making, Wushu Sanda may optimize cognitive–motor control through dual pathways (neuroplasticity and autonomic nervous system regulation), but there is currently a lack of direct evidence on how Sanda training influences this system [13,14]. Therefore, investigating the effects of Sanda on cognitive–motor control not only fills a theoretical gap but also provides a scientific basis for optimizing exercise intervention programs.

While the physical and performance outcomes of Sanda training are well-documented, the underlying neurophysiological adaptations, particularly those pertaining to cognitive–motor control and autonomic regulation, remain largely unexplored. Existing research has primarily focused on external performance metrics, leaving the internal brain and heart dynamics as a “black box.” This study aims to address this gap by employing a multimodal EEG-ECG approach to pioneer a neuro-cardiac perspective on Sanda training.

## 2. Methods

### 2.1. Participants

A total of 20 Sanda athletes and 20 ordinary college students were recruited through public recruitment, all of whom were right-handed males, aged between 18 and 29 years (M ± SD = 23.1 ± 2.4). The inclusion criteria for the Sanda group included having a first-class or higher athlete qualification, with continuous systematic training for ≥5 years, and an average weekly training of ≥10 h in the past 6 months; the control group had no regular exercise habits, and their BMI was matched with that of the Sanda group. Exclusion criteria included having color blindness/color weakness, a history of traumatic brain injury, the presence of mental or cardiovascular diseases, and taking drugs that affect heart rate or the central nervous system. In addition, participants were required not to engage in strenuous exercise and not to consume caffeine or alcohol within 24 h before the experiment. Finally, 1 participant was excluded from each group due to >25% EEG artifacts or >5% missing HRV signals, resulting in 19 valid samples in the Sanda group and 19 in the control group. This study was approved by the Ethics Committee of the Shanghai University of Sport (approval number: 102772024RT018).

### 2.2. Research Design

The experiment adopted a 2 (group: Sanda athletes vs. ordinary college students) × 2 (phase: pre-task rest vs. post-task rest) × 4 (Stroop conditions: cC, cI, iC, iI) three-factor mixed design. The between-group variable was the group, and the within-group variables were phase and Stroop conditions. The dependent variables included (1) behavioral indicators: reaction time, accuracy rate, and conflict adaptation effect [(cI − cC) − (iI − iC)], which measure the reduction in Stroop interference after incongruent trials compared to congruent trials; (2) ERP indicators: average amplitude and difference wave of P600 (550–650 ms) and LSP (700–800 ms); (3) HRV indicators: RMSSD, HF power (ms^2^).

### 2.3. Research Process

The entire experiment was conducted in a soundproof and constant-temperature (25 °C) electromagnetic shielding room, with strictly fixed timing to ensure environmental constancy and comparability of multimodal data. Upon arrival, participants first sat quietly in the rest area for 15 min to adapt to the environment, during which they completed personal information filling and informed consent signing. Subsequently, the experimenter assisted in wearing a 64-channel Ag/AgCl electrode cap (Greentek Pty. Ltd., Hubei, China, positioned according to the international 10–20 system, with grounding and reference at AFz and FCz, respectively) and attaching disposable Ag/AgCl ECG electrodes to form a standard three-lead ECG (RA-LL-LA). The recording equipment was a BrainAMP 64-channel amplifier (Brain Products GmbH, Gilching, Germany) and Brain Vision Recorder 2.0 software. The experimenter briefly explained the experimental process to ensure that participants understood the task requirements and reconfirmed that they had no strenuous exercise, caffeine, or alcohol intake within the last 24 h.

Then, the resting baseline phase began: the lights were dimmed, participants maintained a comfortable sitting posture, closed their eyes to relax but stayed awake, and continuously recorded ECG for 5 min, which served as the pre-task HRV indicator (Pre). Immediately after the baseline, the Stroop-ERP task started. This task adopted the classic color-word Stroop paradigm, consisting of 2 sequences, with 101 trials in each sequence, and the total duration was approximately 13 min. The stimulus materials were the four characters “red, yellow, blue, green”, each presented in four colors. Participants need to determine whether the color of the word is congruent with its meaning. The congruent and incongruent trials each accounted for 50% and were pseudo-randomly arranged to ensure 25 trials for each of the Stroop conditions: cC, cI, iC, and iI. The trial process was as follows: Central fixation point “+” presented for 1000 ms → random blank screen for 300–1000 ms → color-word stimulus for 800 ms → blank screen for 1700 ms. Participants needed to press the “1” key with their right index finger to indicate that the font color was consistent with the word meaning, and the middle finger to press the “2” key to indicate inconsistency. Responses were allowed at any time during the stimulus or blank screen; there was no additional feedback after pressing the key, and the system automatically recorded the key presses and reaction times. Participants were allowed to rest for 30 s between the two sequences, and the experimenter reconfirmed that there was no electrode impedance drift before starting the next sequence.

Immediately after the task was completed, the recovery phase began: the lights were dimmed again, participants maintained the same closed-eye sitting posture as in the baseline phase, and 5 min of ECG was continuously collected as the post-task HRV indicator (Post). Throughout the experiment, TTL synchronization pulses were automatically sent by E-Prime 3.0 to the EEG and ECG acquisition systems at the start and end of each phase, ensuring a time accuracy of <1 ms for cross-modal data to achieve precise coupling of ERP and HRV. After the recovery recording was completed, the experimenter turned off the acquisition system, assisted participants in removing the electrodes and ECG patches, cleaned the skin with alcohol pads, and the entire experiment ended.

### 2.4. Measurement Indicators

This study constructed a measurement system based on three main lines: behavioral performance, neuroelectrophysiology, and cardiac autonomic nervous activity. All indicators followed the collection, processing, and reporting standards jointly recommended by the Society for Psychophysiological Research (SPR) and the European Society of Cardiology (ESC) and ensured the comparability and coupling accuracy of cross-modal data through timestamp synchronization and quality control procedures throughout the experiment.

#### 2.4.1. Behavioral

At the behavioral level, the classic color-word Stroop paradigm was used to record the key-press responses of participants under four sequence conditions (cC, cI, iC, iI) with millisecond precision. Reaction time (RT) was defined as the time interval from the appearance of the stimulus to the correct key press. For each participant, error trials and extreme values (less than 300 ms or greater than the individual mean ± 3 standard deviations) were first excluded, and then the arithmetic mean under each condition was calculated; the accuracy rate (ACC) was the percentage of correct key presses in the valid trials under that condition. To reduce the impact of non-normal distribution, all percentages were transformed using the arcsine square root before entering the subsequent statistical model. The conflict adaptation effect (CAE) was calculated by the formula [(cI − cC) − (iI − iC)], where the values in the brackets were reaction time differences. A larger value of this indicator indicated that an individual had a stronger ability to inhibit and adjust subsequent conflicts after experiencing a conflict.

#### 2.4.2. ERP

At the neuroelectrophysiological level, event-related potential (ERP) data were continuously recorded with a 64-channel Ag/AgCl electrode cap at a sampling rate of 1000 Hz. Offline processing used the EEGLAB/ERPLAB toolchain, including referencing to bilateral mastoids, 0.1–30 Hz zero-phase Butterworth filtering, ICA to remove eye movement and electromyographic artifacts, ±100 μV artifact rejection, segmentation from −200 ms to 1000 ms, and baseline correction from −200 ms to 0 ms. Based on the previous literature and scalp topographic map verification, two-time windows of P600 (550–650 ms) and LSP (700–800 ms) were selected to represent the conflict reanalysis and conflict resolution stages, respectively, with electrode sites at Cz, CPz, Pz and Cz, Pz, Oz, respectively, and the average amplitude (μV) of each participant under each condition was calculated. Prior studies have shown that LSP is often distributed over parietal–occipital/posterior regions during conflict task [15]. P600 has a strong correlation with cognitive control; during stimulus presentation, incongruent trials tend to show greater amplitude compared to congruent trials, reflecting a close relationship with the cognitive control adjustment process [16]. To highlight cognitive control processing, difference waves (incongruent minus congruent) and conflict adaptation ERP indicators, i.e., [(cI − cC) − (iI − iC)], were further generated for cross-level coupling analysis with behavioral CAE. In terms of signal quality, only participants with a proportion of valid trials ≥ 80% after artifact rejection was retained, and the retention rates of the two groups showed no significant difference by independent sample t-test, ensuring comparability between groups.

#### 2.4.3. HRV

At the cardiac autonomic nervous level, standard three-lead ECG (RA-LL-LA) was recorded synchronously at a sampling rate of 1000 Hz with a hardware bandpass of 0.5–150 Hz. R-peak identification was completed by the Kubios Premium 3.5 automatic algorithm, and abnormal beats were checked manually segment by segment and interpolated, with the proportion of abnormal beats controlled within 5%. The time-domain indicator RMSSD (ms) was calculated as the square root of the sum of the squares of adjacent R-R interval differences, reflecting parasympathetic tone; the frequency-domain indicator HF power (ms^2^) was estimated in the 0.15–0.40 Hz frequency band using the Welch periodogram method (256-point Hamming window, 50% overlap), representing pure parasympathetic activity. To control the potential contamination of HF by respiration, participants were prompted to maintain a natural breathing rhythm throughout the experiment, and the Kubios built-in respiratory filter was enabled during the analysis phase for correction. Finally, the differences ΔRMSSD and ΔHF between post- and pre-task were used as individual post-stress recovery indices, with higher values indicating more rapid autonomic nervous recovery. The effective sinus beat sequences in all HRV periods must be >95%; if atrial or ventricular premature beats occurred, the entire segment was excluded and re-collected to ensure data integrity.

### 2.5. Statistical Analysis

All tests in this study were completed in SPSS 26.0, with a unified significance level set to two-tailed α = 0.05, Bonferroni–Holm correction for post hoc multiple comparisons, and partial η^2^ (ηp^2^) for effect size reporting. Firstly, for behavioral and ERP data, a 2 (group: Sanda athletes vs. ordinary college students) × 4 (Stroop conditions: cC, cI, iC, iI) mixed-design repeated measures variance model was established; if Mauchly’s test of sphericity indicated a violation of the assumption (*p* < 0.05), the degrees of freedom and *p*-values corrected by Greenhouse–Geisser were reported. At the behavioral level, the conflict adaptation effect value [(cI − cC) − (iI − iC)] was used as the dependent variable, and at the ERP level, the differential amplitudes of P600 and LSP [(cI − cC) − (iI − iC)] were used as the dependent variables. The same 2 × 4 mixed ANOVA was performed, respectively, to confirm the main effect and interaction effect of the group in the conflict monitoring and conflict resolution stages. Secondly, for HRV recovery indicators, a 2 (group) × 2 (phase: pre-task rest vs. post-task rest) mixed ANOVA was used, with ΔRMSSD and ΔHF as dependent variables, to investigate whether autonomic nervous recovery varies with exercise experience. Thirdly, to explore the neuro-cardiac coupling mechanism, Pearson product–moment correlations between ΔRMSSD, ΔHF, and behavioral conflict adaptation effect, as well as P600 and LSP differential amplitudes, were calculated within each group. To examine whether autonomic recovery mediated the relationship between Sanda experience and cognitive–motor control, a simple mediation model was tested with PROCESS macro (Model 4). Group (Sanda athletes coded 1, controls coded 0) served as the independent variable X; the post-minus-pre change in RMSSD (ΔRMSSD) served as the mediator M; and the behavioral conflict adaptation effect (CAE in ms) served as the dependent variable Y. Age and BMI were entered as covariates. Bias-corrected bootstrap confidence intervals (CI) were computed based on 5000 resamples; a CI that did not straddle zero indicated a significant indirect effect.

## 3. Results

### 3.1. Behavioral Performance

#### 3.1.1. Raw RT and Accuracy

Table 1 summarizes mean RT and ACC for each Stroop condition.

A 2 (Group) × 4 (Condition) mixed ANOVA on RT revealed significant main effects of Group, *F* = 9.25, *p* = 0.004, ηp^2^ = 0.21; Condition, *F* = 15.79, *p* < 0.001, ηp^2^ = 0.31; qualified by a Group × Condition interaction, *F* = 4.21, *p* = 0.049, ηp^2^ = 0.11. Post hoc tests confirmed that athletes were faster than controls in the cC condition (*p* = 0.004), but not in the cI, iC, or iI conditions.

For accuracy, only a Condition main effect emerged, *F* = 4.65, *p* = 0.004, ηp^2^ = 0.11; neither Group (*p* = 0.912) nor the interaction (*p* = 0.781) was significant.

#### 3.1.2. Conflict Adaptation

**Figure 1:**
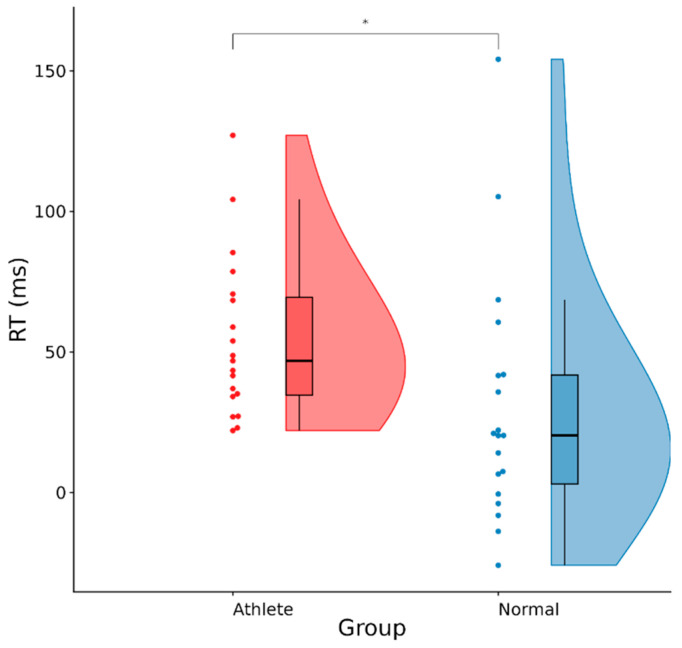
Conflict adaptation effects in athletes and the controls. (* *p* < 0.001).

Table 2 and Figure 1 demonstrate conflict adaptation effects (CAE). RT was significantly greater in athletes (46 ± 21 ms) than controls (21 ± 14 ms), *t* = 4.32, *p* < 0.001.

### 3.2. ERP Results

#### 3.2.1. P600 (550–650 ms, Cz/CPz/Pz)

**Figure 2:**
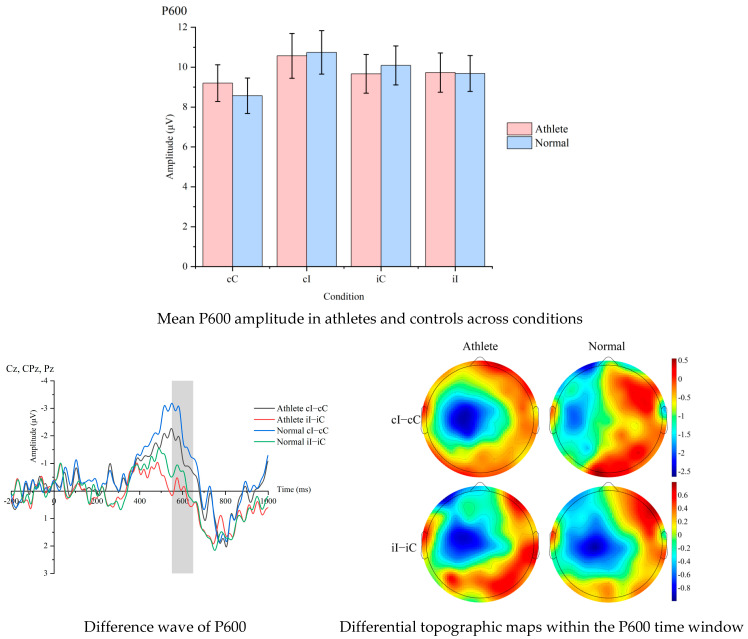
Comparison of P600 difference wave and topographic maps between athletes and non-athletes across conditions.

Mean amplitudes are presented in Table 3. Figure 2 shows the average P600 amplitudes for Sanda athletes and non-athletes under each condition, as well as the difference waves and difference topographies within the P600 time window for the two groups.

A 2 × 4 ANOVA revealed a Condition main effect, *F* = 6.71, *p* = 0.002, ηp^2^ = 0.157, and a Group × Condition interaction for the difference wave CAE, *F* = 4.26, *p* = 0.046, ηp^2^ = 0.106. Athletes exhibited a larger P600 CAE amplitude compared to controls, *t*= 2.44, *p* = 0.020, d = 2.47.

#### 3.2.2. LSP (700–800 ms, Cz/Pz/Oz)

**Figure 3:**
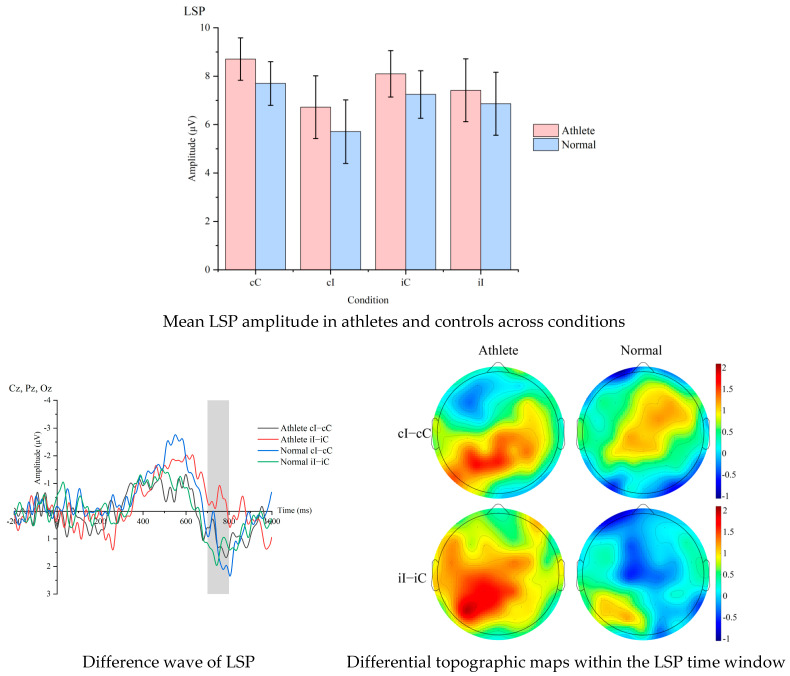
Comparison of LSP difference wave and topographic maps between athletes and non-athletes across conditions.

Table 4 gives mean amplitudes. Figure 3 shows the average LSP amplitudes for Sanda athletes and non-athletes under each condition, as well as the difference waves and difference topographies within the LSP time window for the two groups.

A 2 × 4 ANOVA showed Condition, *F* = 3.51, *p* = 0.018, ηp^2^ = 0.089; Group, *F* = 4.45, *p* = 0.042, ηp^2^ = 0.110; and a Group × Condition interaction for the difference wave CAE, *F* = 4.68, *p* = 0.037, ηp^2^ = 0.115. Athletes’ LSP CAE amplitude exceeded controls, *t* = 2.16, *p* = 0.046, d = 1.52.

#### 3.2.3. Neuro-Behavior Coupling

**Figure 4:**
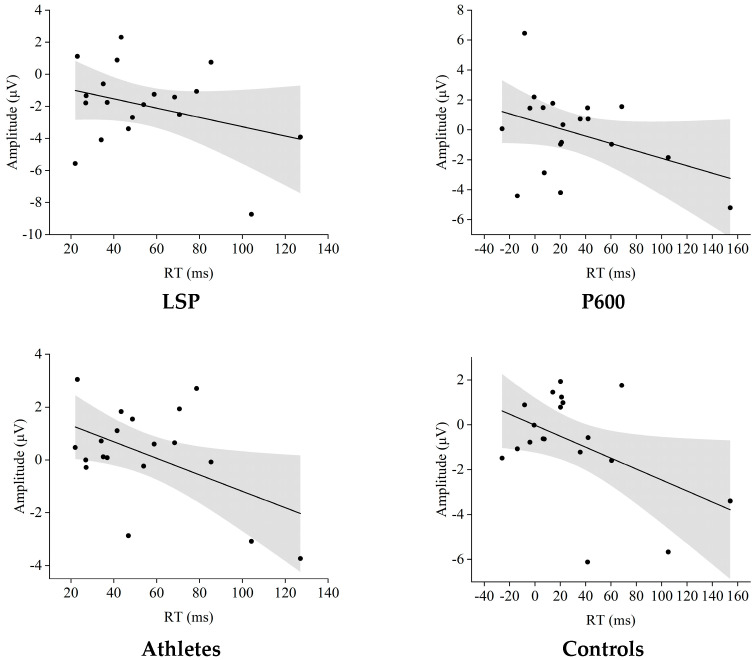
Correlation between amplitude and response-time conflict adaptation effects.

Across both athletes and controls, the relationship between neural conflict signatures and behavioral conflict adaptation exhibited a complex pattern. For the P600 component, both groups showed a negative correlation—athletes at *r* = −0.33 and controls at *r* = −0.39; however, these associations did not reach conventional significance (*p* = 0.173 and 0.101, respectively). It is important to note that no statistically significant association was found for the P600 component. In contrast, the LSP provided a clearer bridge between brain and behavior. Here, the negative correlations were not only stronger—*r* = −0.49 for athletes and *r* = −0.47 for controls—but also statistically reliable (*p* = 0.033 and 0.043) (Figure 4). 

### 3.3. Heart-Rate Variability

#### 3.3.1. RMSSD and HF Power

Table 5 presents pre-task, post-task, and Δ values.

A 2 × 2 (Group × Phase) ANOVA on RMSSD revealed a Phase × Group interaction, *F* = 5.88, *p* = 0.020, ηp^2^ = 0.141. Athletes demonstrated a larger vagal rebound than controls, *t* = 2.42, *p* = 0.021, d = 1.64.

A 2 × 2 (Group × Phase) ANOVA on HF power revealed a Phase × Group interaction, *F* = 4.93, *p* = 0.033, ηp^2^ = 0.120. Athletes demonstrated a higher HF than controls, *t* = 2.22, *p* = 0.032, d = 1.59.

#### 3.3.2. HRV–ERP Coupling

In the athlete group, ΔRMSSD showed a significant positive correlation with behavioral conflict adaptation RT (*r* = 0.45, *p* = 0.048) and a significant negative correlation with LSP CAE amplitude (*r* = −0.42, *p* = 0.009); all remaining correlations were non-significant (ps > 0.05). These correlation results are detailed in Table 6. In the control group, none of the correlations reached significance (ps > 0.16).

### 3.4. Mediation Analyses

The mediation results are summarized in Table 7. The total effect of group on CAE was significant, c = 24.8, 95% CI [12.3, 37.2]. When ΔRMSSD was entered into the model, the direct effect dropped but remained significant, c′ = 15.9, 95% CI [3.9, 27.9]. Crucially, the indirect effect through ΔRMSSD was also significant, ab = 8.9, 95% CI [2.7, 16.1]. Approximately 36% of the total effect of Sanda experience on cognitive–motor control was carried by enhanced vagal recovery.

## 4. Discussion

### 4.1. Analysis of Behavioral Performance Results

The results of this study indicate that long-term Sanda training significantly improved athletes’ reaction time (RT) in the Stroop task and demonstrated stronger conflict adaptation ability (CAE) under conflict conditions (such as cI and iI), while accuracy (ACC) showed no significant difference with the control group. This finding aligns with previous research on combat sports, which suggests that high-intensity, competitive physical activities can optimize an individual’s cognitive–motor control abilities, particularly in conflict monitoring and response inhibition [16,17]. Previous studies have also observed that combat athletes exhibit shorter reaction times in tasks requiring rapid decision-making, but their accuracy rates are comparable to those of the general population, suggesting that such sports may enhance performance by improving neural efficiency (e.g., reducing redundant processing) without sacrificing accuracy [18]. Furthermore, the RT advantage of Sanda athletes under conflict conditions in this study further supports the ‘sport-specific transfer’ hypothesis, which posits that long-term specialized training can shape cognitive–motor control patterns highly aligned with task demands [19]. The larger P600 and LSP amplitudes observed in athletes may reflect enhanced and more specialized neural engagement for conflict processing, rather than merely increased resource expenditure. This finding not only provides empirical support for the cognitive benefits of Sanda training but also offers theoretical grounds for using sports interventions to improve conflict processing abilities, emphasizing that the synergistic interaction between specialized neural engagement and autonomic nervous system regulation may be a key mechanism underlying sports-induced cognitive optimization.

Additionally, the study found that martial arts Sanda athletes demonstrated a significant advantage in conflict adaptation effect (CAE), with their CAE amplitude being significantly greater than that of the general control group. This finding is consistent with previous research on the cognitive control abilities of combat athletes, which suggests that long-term high-intensity combat training can significantly enhance an individual’s dynamic regulatory abilities in conflict tasks [20,21]. Previous studies have shown that combat athletes exhibit stronger conflict monitoring and adaptive regulation abilities in tasks requiring rapid adjustment of response strategies, which aligns with the enhanced CAE observed in this study, further validating the promotional effect of athletic training on conflict adaptation abilities [22]. Notably, the Stroop task design in this study effectively distinguished performance differences under different conflict conditions, providing more refined evidence for the association between athletic training and cognitive control. These findings not only reinforce the notion that athletic experience shapes higher-order cognitive functions but also provide important references for developing cognitive enhancement programs based on athletic interventions. The results support the use of CAE as a sensitive indicator for assessing the impact of athletic training on cognitive function, and future studies may further explore the selective effects of different athletic types on specific cognitive subfunctions.

### 4.2. Analysis of ERP Results

This study found that in the P600 time window, Sanda athletes exhibited stronger CAE-related neural activity. This result is consistent with previous findings on cognitive control in athletes, namely that specialized training can enhance the brain’s ability to monitor conflicting information early on [23]. Previous studies have shown that the P600 component reflects an individual’s allocation of attentional resources and re-evaluation process in response to conflict signals. The larger P600 amplitude observed in athletes in this study further supports the hypothesis that athletic training may enhance conflict monitoring efficiency by strengthening functional connectivity within the prefrontal-parietal network [24]. This finding provides new neurophysiological evidence for understanding how athletic training optimizes higher-order cognitive functions and also offers a theoretical basis for developing cognitive assessment methods for athletes based on P600 indicators.

The results showed that in the LSP time window, Sanda athletes also exhibited more significant CAE-related neural activity. This finding aligns with some previous research on the effects of athletic training on late-stage cognitive processing. Previous studies have indicated that the LSP component is closely related to conflict resolution and response inhibition processes. The larger LSP amplitude observed in athletes in this study suggests that long-term Sanda training may enhance behavioral performance by optimizing the brain’s late-stage processing strategies for conflict information [25]. Notably, this finding, together with the P600 findings, forms a complete evidence chain suggesting that athletic training may enhance overall cognitive performance by separately enhancing the neural processing efficiency of the early conflict monitoring and late conflict resolution stages [26].

This study observed a significant negative correlation between the LSP component and behavioral CAE, providing direct evidence for understanding how athletic training shapes the brain–behavioral synergy mechanism. Previous studies on neural efficiency have generally found that well-trained individuals exhibit more streamlined neural activity patterns, consistent with the results of this study. Notably, this neural–behavioral coupling relationship was more pronounced in the LSP window than in the P600 window, suggesting that the late conflict resolution process may be a critical component through which motor training influences behavioral performance [27]. This finding not only deepens our understanding of the relationship between motor training and cognitive function but also provides important insights for developing athlete cognitive assessment protocols based on multi-time-window ERP analysis.

### 4.3. Analysis of Heart Rate Variability Results

This study found that in terms of HRV indicators, Sanda athletes demonstrated stronger vagal recovery capacity, specifically manifested as significantly higher ΔRMSSD and ΔHF compared to the control group. This result is consistent with previous research findings on the autonomic nervous system regulatory advantages of athletes, namely that long-term athletic training can enhance the rapid recovery capacity of the parasympathetic nervous system [28]. Previous studies have shown that elite athletes often exhibit faster HRV recovery after stressful tasks, which is consistent with the greater vagal rebound observed in athletes in this study [29]. This finding provides important evidence for understanding how exercise training promotes cognitive recovery by improving autonomic nervous system function and also offers an objective indicator for assessing athletes’ physiological adaptation status. Additionally, the results showed a significant positive correlation between ΔRMSSD and behavioral CAE, which aligns with some previous studies on the relationship between autonomic nervous system function and cognitive performance. Previous research has indicated that individuals with higher vagal tone typically perform better in tasks requiring cognitive flexibility, and this study further confirms that this association is particularly pronounced in professional athletes. Notably, this correlation only reached statistical significance in the athlete group, suggesting that long-term Sanda training may have strengthened the coupling relationship between autonomic nervous system regulation and cognitive function [30]. This result provides a theoretical basis for developing athlete cognitive function assessment methods based on HRV indicators.

On the other hand, this study found a significant negative correlation between ΔRMSSD and LSP amplitude, providing new evidence for understanding neuro-cardiac coordination mechanisms. Previous studies on brain–heart interaction have generally observed functional connections between vague nerve activity and specific EEG components. This study is the first to confirm this association pattern in an athletic population. Notably, this coupling relationship is primarily manifested in the late conflict resolution process (LSP) rather than early conflict monitoring (P600), suggesting that autonomic regulation may primarily optimize behavioral performance by influencing the neural efficiency of conflict resolution [31]. This finding provides important clues for establishing a more comprehensive model of motor–cognitive–autonomic integration. Previous studies have suggested that autonomic nervous system function may be an important pathway through which exercise influences cognition [32]. This study provides indirect evidence for this hypothesis. However, it is important to note that due to the cross-sectional design of this study, causal inferences cannot be definitively established. The mediating effect observed is consistent with a neuro-cardiac coupling hypothesis, but alternative explanations or bidirectional relationships remain possible. Future longitudinal or intervention studies are necessary to clarify the causal pathways involved. Nonetheless, these findings highlight the potential value of incorporating autonomic nervous system monitoring—such as respiratory regulation training—into future interventions aimed at optimizing cognitive performance and recovery in athletes.

**Limitations and Research Prospects**: This study has several limitations that should be considered. First, the cross-sectional nature of our design cannot definitively rule out the potential influence of self-selection bias, where individuals with pre-existing advantages in neuro-cardiac function might be more likely to excel and persist in Sanda training. Therefore, the observed associations may reflect a combination of training effects and innate predisposition. Second, the sample size, while consistent with many neurophysiological studies, limits the generalizability of our findings. Future research should employ longitudinal or intervention designs starting with novice participants to establish causality. Furthermore, expanding the sample size and including athlete groups from other sports disciplines would help determine the specificity of the observed effects. Finally, utilizing mobile neuroimaging and physiological sensors in ecologically valid settings, such as simulated competitions, is crucial for understanding how these neuro-cardiac mechanisms translate to real-world performance.

## 5. Conclusions

This study confirmed through multimodal experiments that long-term martial arts Sanda training can significantly improve cognitive–motor control abilities. This effect is achieved through three synergistic mechanisms: at the behavioral level, it manifests as improved conflict adaptation abilities; at the neural level, it is reflected in enhanced conflict monitoring (P600) and resolution (LSP) efficiency; and at the autonomic nervous system level, it is manifested as faster vagal nerve recovery (HRV). This study systematically reveals for the first time the complete pathway through which Sanda training optimizes cognitive function via the “neuro-cardiac” coupling mechanism. While the generalizability of these findings to other types of sports training or non-athlete populations requires further investigation, our results provide important evidence for the theoretical development and practical application of exercise science and also open new avenues for the design of exercise intervention programs for cognitive dysfunction.

## Figures and Tables

**Table 1:** Mean reaction time (ms) and accuracy (%) by condition and group.

Condition	Athletes RT (ms)	Controls RT (ms)	*p*	ηp^2^	Athletes ACC (%)	Controls ACC (%)	*p*	ηp^2^
cC	595.82 ± 59.15	669.92 ± 67.26	0.004	0.21	95.63 ± 4.02	94.74 ± 5.69	0.912	0.00
cI	667.94 ± 53.22	725.75 ± 89.88	0.30	0.03	94.86 ± 3.92	95.59 ± 4.13	0.781	0.00
iC	621.76 ± 58.03	674.61 ± 71.97	0.36	0.02	92.90 ± 5.11	93.69 ± 6.74	0.701	0.00
iI	639.04 ± 37.12	700.53 ± 76.85	0.24	0.04	96.43 ± 3.88	96.38 ± 4.71	1.000	0.00

**Table 2:** Conflict adaptation effects (RT, ms).

Index	Athletes (M ± SD)	Controls (M ± SD)	*t*	*p*
CAE	46 ± 21	21 ± 14	4.32	<0.001

**Table 3:** P600 mean amplitudes (μV) and follow-up contrasts.

Condition	Athletes (M ± SD)	Controls (M ± SD)	F(1,36)	p(Interaction)	ηp^2^
cC	9.20 ± 4.02	8.57 ± 3.88	0.11	0.74	0.003
cI	10.57 ± 4.89	10.74 ± 4.74	0.02	0.88	0.001
iC	9.67 ± 4.23	10.09 ± 4.26	0.09	0.76	0.003
iI	9.73 ± 4.29	9.68 ± 3.92	0.001	0.97	<0.001

**Table 4:** LSP mean amplitudes (μV) and follow-up contrasts.

Condition	Athletes (M ± SD)	Controls (M ± SD)	F(1,36)	p(Interaction)	ηp^2^
cC	8.71 ± 3.82	7.70 ± 3.94	0.67	0.42	0.018
cI	6.72 ± 5.65	5.71 ± 5.72	0.46	0.50	0.013
iC	8.10 ± 4.17	7.25 ± 4.28	0.49	0.49	0.013
iI	7.42 ± 5.66	6.86 ± 5.66	0.11	0.74	0.003

**Table 5:** HRV indices before and after the Stroop task with recovery Δ-values.

Index	Phase	Athletes (M ± SD)	Controls (M ± SD)	*p*	ηp^2^	ΔAthletes (M ± SD)	ΔControls (M ± SD)	*p*	d
RMSSD (ms)	Pre-task	42.3 ± 8.9	41.5 ± 9.1	0.020	0.141	+12.4 ± 6.1	+3.1 ± 5.3	0.021	1.64
	Post-task	54.7 ± 9.8	44.6 ± 8.4	—	—	—	—	—	—
HF (ms^2^)	Pre-task	687 ± 203	672 ± 198	0.033	0.120	+145 ± 72	+41 ± 58	0.032	1.59
	Post-task	832 ± 221	713 ± 185	—	—	—	—	—	—

Note: Δ = Post-task − Pre-task. P-statistics refer to the Phase × Group interaction from the 2 × 2 mixed ANOVA.

**Table 6:** Pearson correlations between autonomic recovery indices (ΔRMSSD, ΔHF) and neural–behavioral markers of conflict adaptation in Sanda athletes and controls.

Variable Pair	Group	*r*	*p*
**ΔRMSSD × Behavioral CAE RT**	Athletes	0.45	0.048
	Controls	0.31	0.129
**ΔRMSSD × P600 amplitude**	Athletes	−0.22	0.301
	Controls	−0.15	0.472
**ΔRMSSD × LSP amplitude**	Athletes	−0.42	0.009
	Controls	−0.28	0.166
**ΔHF × Behavioral CAE RT**	Athletes	0.32	0.098
	Controls	0.21	0.311
**ΔHF × P600 amplitude**	Athletes	−0.18	0.401
	Controls	−0.11	0.608
**ΔHF × LSP amplitude**	Athletes	−0.33	0.076
	Controls	−0.20	0.337

**Table 7:** Mediation results from PROCESS Model 4 (Outcome: CAE).

Effect	Path	Coeff.	SE	95% CI	*p*
Direct (X→M)	a	9.30	1.80	[5.70, 12.90]	<0.001
Direct (M→Y)	b	0.96	0.30	[0.40, 1.60]	0.003
Total (X→Y)	c	24.80	6.20	[12.30, 37.20]	<0.001
Direct (X→Y→M)	c′	15.90	6.00	[3.90, 27.90]	0.012
Indirect (via M)	ab	8.90	3.40	[2.70, 16.10]	

## Data Availability

The original contributions presented in this study are included in the article. Further inquiries can be directed to the corresponding author.
